# TLR4 Signaling by Heme and the Role of Heme-Binding Blood Proteins

**DOI:** 10.3389/fimmu.2020.01964

**Published:** 2020-08-27

**Authors:** Sabina Janciauskiene, Vijith Vijayan, Stephan Immenschuh

**Affiliations:** ^1^Department of Pulmonology, Biomedical Research in Endstage and Obstructive Lung Disease Hannover (BREATH), Member of the German Center for Lung Research (DZL), Hannover Medical School, Hanover, Germany; ^2^Institute for Transfusion Medicine and Transplant Engineering, Hannover Medical School, Hanover, Germany

**Keywords:** heme, heme-binding proteins, hemopexin, hemolysis, inflammation, TLR4

## Abstract

Toll-like receptors (TLRs), also known as pattern recognition receptors, respond to exogenous pathogens and to intrinsic danger signals released from damaged cells and tissues. The tetrapyrrole heme has been suggested to be an agonist for TLR4, the receptor for the pro-inflammatory bacterial component lipopolysaccharide (LPS), synonymous with endotoxin. Heme is a double-edged sword with contradictory functions. On the one hand, it has vital cellular functions as the prosthetic group of hemoproteins including hemoglobin, myoglobin, and cytochromes. On the other hand, if released from destabilized hemoproteins, non-protein bound or “free” heme can have pro-oxidant and pro-inflammatory effects, the mechanisms of which are not fully understood. In this review, the complex interactions between heme and TLR4 are discussed with a particular focus on the role of heme-binding serum proteins in handling extracellular heme and its impact on TLR4 signaling. Moreover, the role of heme as a direct and indirect trigger of TLR4 activation and species-specific differences in the regulation of heme-dependent TLR4 signaling are highlighted.

## Introduction

Toll-like receptors (TLRs) recognize invading pathogens and are essential sensors and regulators of the innate immune system (1, 2). Bacterial, fungal, and viral infections activate various TLRs that play a role in host defense but may also cause sepsis and tissue injury. Stimulation of TLRs by their respective specific ligands initiates signaling cascades that mediate activation of transcription factors and secretion of pro-inflammatory molecules (1, 2). For instance, TLR4 is stimulated by the prototypical pro-inflammatory bacterial wall compound lipopolysaccharide (LPS), also known as endotoxin (3). More recently, other compounds have been described to interact and stimulate TLR4 including hyaluronic acid, the dust mite protein Der p 2, nickel and various endogenous molecules released from injured cells, that are collectively termed danger-associated molecular patterns (DAMPs) (4–7). In particular, the red blood cell-derived product heme has been implicated in TLR4 signaling and has been proposed to be a DAMP that affects inflammatory responses in a variety of pathophysiological conditions (8–15). Heme is an iron-containing tetrapyrrole with important functions in various biological processes as a prosthetic moiety of hemoproteins in its covalent or non-covalent bound form (16, 17). For example, in hemoglobin and myoglobin, heme is used for oxygen transport and storage, whereas in cytochromes it is involved in electron transport, and generation of energy. Heme is also important for enzymes such as cyclooxygenase-2, nitric-oxide synthase-1, NADPH oxidases, catalases, and peroxidases (16, 18). In contrast, non-protein bound heme, also termed “free” heme, can be harmful and cause pro-oxidant, pro-inflammatory, and cytotoxic effects as previously reviewed elsewhere (12, 13, 19, 20). Additionally, heme can mediate the recruitment of leukocytes, platelets, and red blood cells to the vascular endothelium. Many of the pro-inflammatory effects of heme have been associated with activation of TLR4 signaling, as initially demonstrated in macrophages (10). However, TLR4 signaling by heme appears to involve highly complex regulatory mechanisms, which are dependent on the applied models and experimental conditions (15, 21). For example, conflicting findings on potential heme-dependent pro-inflammatory effects have been reported in kidney injury models applying TAK-242, a specific inhibitor of TLR4 signaling, and TLR4 knockout mice (22–25). Hence, mechanistic details on how heme may mediate its pro-inflammatory regulation through direct or indirect interactions with TLR4 are not fully understood. In this review, the complex relationships between heme and TLR4 are discussed with a particular focus on the role of serum heme-binding proteins (HBPs).

## Direct Activation of TLR4 Signaling by Heme

**Figure 1:**
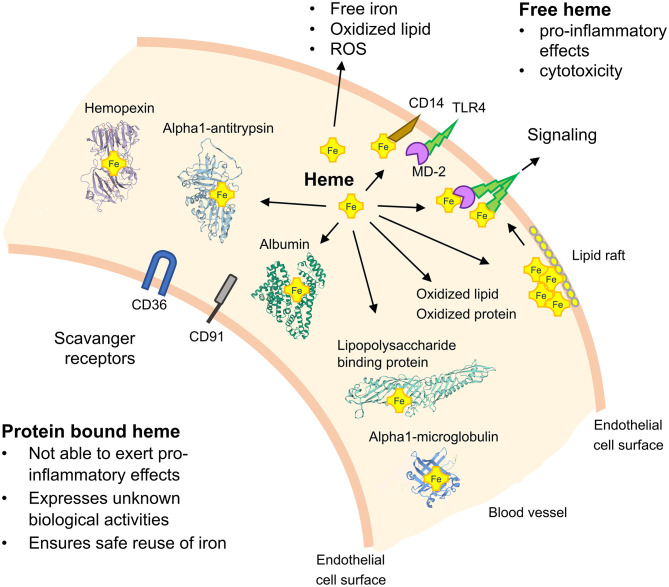
Schematic on TLR4 signaling by heme and the role of heme-binding blood proteins. TLR4 activation depends on the serum protein lipopolysaccharide (LPS)-binding protein, the co-receptor CD14 and myeloid differentiation protein-2 (MD-2) for bacterial recognition. Free heme can activate TLR4-mediated signaling and soluble CD14 has been found to be critical for the regulatory effects of heme. Heme-binding proteins (HBPs) including hemopexin (Hx), albumin, alpha1-microglobulin (A1M), and alpha1-antitrysin (AAT) play critical roles for neutralization of heme and potential heme-dependent interactions with TLR4 to activate TLR4 signaling. The potential role of lipid rafts in TLR4 signaling and scavenger receptors, such as CD36 and CD91, in mediating cellular effects of heme are depicted. *ROS*, reactive oxygen species.

The mechanistic basis of how TLR4 signaling may be activated by heme has been primarily studied in mouse models with genetic TLR4 deficiency and with small molecule inhibitors of TLR4. For example, it has been demonstrated that treatment of TLR4-deficient macrophages with purified exogenous heme fails to induce expression of pro-inflammatory cytokines (10) and activation of the inflammasome (26). Moreover, inflammatory activation of the endothelium by heme has been found to be counter-acted in TLR4^−/−^ mice and by administration of TAK-242 (27). Interestingly, in studies with human embryonic kidney 293 cells, heme, and LPS applied together expressed additive effects suggesting that they activate TLR4 by different mechanisms (28). Although such findings support a role of heme in direct TLR4 signaling, an activation site for heme-binding in this receptor is still elusive. As efficient TLR4-dependent cell activation by LPS requires the complex interplay of TLR4 with CD14, myeloid differentiation protein-2 (MD-2) and the serum protein lipopolysaccharide binding protein (LBP) (29) it is likely that cooperation of these proteins is also critically involved in heme-dependent TLR4 signaling (Figure 1). Notably, a heme activation site has recently been identified in human MD-2 which appears to play a critical regulatory role in TLR4 signaling by heme (30).

## Indirect Regulation of TLR4 Signaling by Heme

TLR4 ligands other than LPS can mediate TLR4 signaling independent of direct interactions with the receptor. For example, both, hyaluronic acid and the dust mite allergen Der p 2, have been demonstrated to induce TLR4 signaling indirectly (31, 32). Similarly, accumulating evidence indicates that certain pro-inflammatory heme effects may also be independent of direct heme-binding to TLR4.

### Generation of Reactive Oxygen Species (ROS)

Pro-oxidant properties of free heme can cause the generation of ROS via the Fenton reaction of Fe(II) and H_2_O_2_ [reviewed elsewhere (14, 20, 33)]. As activation of TLRs and generation of ROS can be complementary in settings of so-called oxidative stress (34), it is likely that heme-induced ROS generation may also indirectly activate TLR4 signaling (Figure 1). It should be noted that ROS can rapidly oxidize phospholipids, which in turn initiate pro-inflammatory responses via TLR2 and/or TLR4. Independently, an inhibition of the oxidized 1-palmitoyl-2-arachidonoyl-sn-glycero-3-phosphocholine (OxPAPC)-induced pro-inflammatory effects has been reported after down-regulation of TLR4 either by the antagonist eritoran or by antisense nucleotides (35–37).

### Lipid Oxidation

Oxidized low-density lipoproteins reportedly cause activation of TLR4 (38) and binding of heme can rapidly bind to and oxidize lipoproteins in the serum (39, 40) (Figure 1). As binding of heme to lipoproteins occurs faster than that to serum HBPs such as hemopexin (Hx) and albumin, it is conceivable that oxidized lipoproteins can induce TLR4-mediated inflammatory signaling and expression of inflammatory cytokines. Yet, depending on the tissue, these inflammatory effects may contribute to arteriosclerosis, rheumatic diseases, and others (33, 40, 41).

### Interaction With Lipid Raft-Associated Proteins

Due to its lipophilic nature, heme can form aggregates and interact with the hydrophobic phospholipid bilayer in lipid membranes affecting TLR4 signaling (42). Membrane lipid rafts are dynamic cellular assemblies of saturated sphingolipids, cholesterol, and selected proteins (43). There are some transmembrane proteins located in lipid rafts including CD44 and CD36, both of which are involved in TLR4 signaling. Ample data indicate that TLR4 and accessory proteins can associate with lipid rafts and that TLR4-raft association is stimulated by bacterial LPS (44). Depending on the TLR4 ligand, different co-receptors can be involved. For instance, the ability of LPS to activate TLR4 depends on CD14, a glycophosphatidylinositol-anchored protein and co-receptor of MD-2 for LPS recognition (45), which may also control internalization of heme via TLR4 (10). Interestingly, soluble TLR4 co-receptor CD14 has recently been reported to mediate pro-inflammatory effects of heme in a whole blood model (46).

### Disruption of Lipid-Rafts

Extraction or sequestration of cholesterol with cyclodextrin or nystatin has been shown to disturb clustering of TLR4 and accessory proteins in rafts and to inhibit LPS-induced TNF-α production (47). According to recent reports, naturally high content of cholesterol in sickle and normal red blood cells provides protection against free heme-induced oxidative stress and membrane damage during normal and hemolytic conditions (48). Because cholesterol depletion affects lipid raft assembly, membrane trafficking, and TLR signaling, we speculate that free heme or specific heme-HBP complexes may have modulatory effects on TLR4 signaling via lipid rafts. Thus, we hypothesize that heme, depending on its conformational state, might be incorporated into rafts of the plasma membrane, affect lipid raft fluidity, polarity, thickness, and tension-properties, which, in turn, may influence recruitment (assembly) of TLRs and signaling. Thus, via unspecific hydrophobic interactions with lipid rafts, heme alone or in complex with HBPs may affect TLR4 signaling (Figure 1).

In summary, heme may mediate TLR4 activation via various indirect mechanisms including production of ROS, oxidation of lipoproteins, and modulation of lipid rafts in cell membranes.

## Heme Interactions With Serum Heme-Binding Proteins and Role in TLR4 Signaling

Heme toxicity and its pro-inflammatory effects have been demonstrated in experimental disease models like sickle cell disease (SCD), malaria, sepsis, atypical hemolytic uremic syndrome, arteriosclerosis, or ischemia-reperfusion injury (27, 41, 49–52). The damaging effects of free heme can be blocked by intracellular factors like heme oxygenases and ferritin, and extracellular factors such as various plasma proteins, respectively (Figure 1). Only if both intra- and extracellular defense mechanisms are overwhelmed, cellular toxicity arises (12, 33, 53). Independent reports have provided evidence that neutralization of free heme via Hx, the serum protein with the highest known heme-binding affinity (Kd < 10^−12^ in humans), counteracted the detrimental effects of heme (42, 54–57). However, serum concentrations of Hx are low (about 0.6–1.2 g/L), and in conditions of severe hemolysis (55) decreased systemic levels of Hx might not be sufficient to neutralize larger amounts of free heme. Therefore, other plasma proteins including albumin, alpha-1-microglobulin (A1M), and alpha1-antitrypin (AAT) appear to be also involved in binding and neutralization of free heme (12, 33, 58, 59). Although albumin binds heme with an affinity about 100-fold lower than Hx, the high concentration of albumin in serum (35–53 g/L) might compensate any potential deficiency in Hx. This, in part, may explain beneficial effects of albumin infusion to individuals with severe sepsis (60) and malaria (61, 62). Notably, albumin is a negative acute phase protein in humans and it is conceivable that during severe inflammatory conditions, when the heme-neutralizing capacity of albumin decreases, other acute-phase proteins such as AAT will participate. AAT is a HBP with binding affinity similar to albumin (59) and it has previously been demonstrated that AAT markedly reduces free heme neutrophil-activating effects, including the production of ROS (63). Serum HBPs not only bind and neutralize free heme with different binding affinities, but may also acquire novel biological activities via specific interactions with heme (64–66). For example, the HBPs Hx and A1M have recently been shown to exhibit differential heme transporter functions and are reciprocally regulated during SCD. While Hx directs heme to the liver and mediates its hepatic up-take via the scavenger receptor low-density lipoprotein receptor-related protein-1 (LRP1, synonymous with CD91) (67, 68), A1M directs heme to the kidney where it may cause detrimental effects including acute kidney injury (69). Finally, it has been found that the interplay of immunoglobulins with heme may alter their binding affinity for bacterial antigens (70).

The question, which form(s) of protein-associated heme is/are inert or biologically active *in vivo* remains open. For instance, high concentrations of albumin-associated heme in the presence of serum failed to induce inflammatory responses in endothelial cells and macrophages (21). Likewise, the local and systemic exposure to protein-associated heme did not induce inflammatory gene expression in mouse models. Heme-mediated signaling via NF-kB only occurred in serum-free conditions in cell cultures of macrophages (21). These findings imply that only the complete absence of serum proteins may allow TLR4 interactions of free heme or specific heme-HBP complexes which, in turn, activate pro-inflammatory pathways. Thus, direct heme-mediated TLR4 signaling appears to be unlikely in relevant clinical conditions, because levels of “free” heme *in vivo* appear to be orders of magnitude below those conditions applied *in vitro* to cause pro-inflammatory effects.

In conclusion, pro-inflammatory effects of heme are critically dependent on heme interactions with serum HBPs, which can largely vary in different pathophysiological settings.

## Heme as a Second Hit for TLR4 Activation

Cell-free hemoglobin and heme derived from lysed red blood cells have been reported to synergize with the pro-inflammatory effects of TLR4 agonists in culture models of mouse macrophages (11). These findings suggest that free heme may substantially aggravate inflammatory responses in settings of bacterial or viral infections with simultaneous intravascular hemolysis. Due to the difficulties in determining the biologically relevant concentrations of free heme, the mechanisms that mediate the synergism of heme with different TLR agonists are unclear. Independently, free heme has been demonstrated to synergistically activate the NOD-like receptor family pyrin domain containing 3 (NLRP3) inflammasome in LPS-primed macrophages (26) and endothelial cells (71). The NLRP3 inflammasome is a multimeric protein complex comprising a sensor, an adaptor and the zymogen procaspase-1, which leads to activation of caspase-1 and release of the pro-inflammatory interleukins, IL-1β, and IL-18 (72). Heme activates the NLRP3 inflammasome leading to IL-1β production by peritoneal macrophages and in human endothelial cells, but this effect of heme is lost in NLRP3-deficient mice. Finally, free heme may contribute to the inflammatory activation of the endothelium via complement activation as demonstrated in various experimental models of intravascular hemolysis (51, 73). These studies have also provided experimental evidence that free heme may be an important second signal for pre-existing conditions of pro-inflammatory endothelial activation to further escalate the inflammatory vascular damage in disorders such as SCD and atypical hemolytic uremic syndrome (74).

In summary, heme may synergize with a variety of pro-inflammatory agonists to aggravate activation of TLR4 and inflammation.

## Species-Specific Differences of Heme-Dependent TLR4 Signaling in Inflammation

Because heme interactions with TLR4 have largely been studied in rodent models, the extent to which these models apply to human conditions is very important. Due to the specific pathogens encountered by mice and humans, various aspects in the innate and adaptive immune systems are different between these two species (75). Thus, human and murine responses to TLR4 activation have some similarities, but also profound differences (76). For example, Akashi et al. reported that the lipid moiety of endotoxin, lipid A, acts agonistically on mouse, but not on human TLR4/MD-2 (77), which has more recently also been confirmed in structural studies on the TLR4/MD-2 complex (78). It is also important to point out that murine and human TLR4 share 67–71 and 79–81% similarity at the nucleotide and amino acid levels, respectively (79, 80). Amino acid similarity between the mouse and human TLR4 sequences is 62% in the extracellular domain, 70% in the transmembrane domain, and 83% in the cytoplasmic domain (81). In mice, as in humans, cells of myeloid origin such as monocytes, macrophages, microglia, and granulocytes exhibit the highest levels of TLR4 expression. However, in sharp contrast to human macrophages and monocytes, which increase TLR4 expression in response to LPS, mouse peritoneal macrophages, and neutrophils decrease TLR4 expression after LPS challenge (82). Schroder et al. reported differences in the gene regulation of human and murine macrophages following LPS stimulation. Although various genes targeted by TLR4 signaling are more rapidly induced by LPS in human than in mouse macrophages, several negative feedback loops of the TLR4 pathway are differentially regulated in mouse macrophages (76). Existing knowledge suggests that rabbits and swine may be closer to humans than mice concerning TLR4 sequences and function. In fact, humans, swine, and rabbits are sensitive to LPS with physiological changes induced by a dose at nonograms per kilogram whereas mice are highly resistant to LPS with physiological changes induced by a dose at milligrams per kilogram (83, 84).

Given these above mentioned variations, it does not come as a surprise that mouse and human TLR4 signaling in response to free or HBP-bound heme appears to exhibit substantial differences (85). Moreover, TLR4 activation by LPS has also been found to cause opposing effects on the regulation of intracellular heme levels and heme oxygenase-1 expression in murine and human macrophages (86, 87). Furthermore, determinations of Hx in mouse models of endotoxemia, burn wound infections and peritonitis as compared to those in patients with sepsis and severe burns revealed that systemic levels of this HBP increased above baseline in each murine model, but decreased in comparable human inflammatory conditions (88). Hence, Hx is induced during the so-called acute phase response in rodents, but not in human (33, 89, 90). Another example is AAT (59), because plasma baseline concentrations of AAT in mice are about four times higher than in human plasma (normal levels in human plasma 1.3–2 g/L) (91), which may be important for neutralization and/or susceptibility to free heme toxicity. Thus, species-specific profiles of serum proteins may determine principle differences between mouse and human as shown for defense strategies against bacterial infections (92). Overall, mice have evolved in a different environment to humans, have a markedly lower body weight and have significantly shorter lifespans and, therefore, it is worth considering that the response to heme in mice may not occur in precisely the same way in humans (75, 93). Consequently, TLR4 activation in humans by heme is different from that in mouse models and such evolutionary differences need to be taken into account when translating findings from mouse disease models into human clinical applications.

In conclusion, species-specific differences between mouse and human appear to also apply to heme- and HBP-dependent pathways in TLR4 signaling.

## Conclusions and Outlook

The regulatory role of heme in TLR4 signaling might be dependent on direct and indirect interactions. In particular, the interplay of heme with specific serum HBPs appears to play a major modulatory role in inflammatory conditions. Due to species-specific differences in heme-dependent TLR4 signaling findings from mouse models in experimental inflammatory diseases need to be carefully interpreted when translated to clinical settings. A major challenge will be to establish methods for determination of free heme in physiological and pathophysiological settings to allow a better understanding of the link between heme and the innate immune system.

## Author Contributions

SJ, VV, and SI planned and wrote the manuscript. All authors contributed to the article and approved the submitted version.

## Conflict of Interest

The authors declare that the research was conducted in the absence of any commercial or financial relationships that could be construed as a potential conflict of interest.
